# Increased Risk of Influenza Infection During Cold Spells in China: National Time Series Study

**DOI:** 10.2196/55822

**Published:** 2024-08-13

**Authors:** Haitao Wang, Mengjie Geng, Tamara Schikowski, Ashtyn Tracey Areal, Kejia Hu, Wen Li, Micheline de Sousa Zanotti Stagliorio Coelho, Paulo Hilário Nascimento Saldiva, Wei Sun, Chengchao Zhou, Liang Lu, Qi Zhao, Wei Ma

**Affiliations:** 1 Department of Epidemiology, School of Public Health, Cheeloo College of Medicine, Shandong University, Jinan, China; 2 Shandong University Climate Change and Health Center, Jinan, China; 3 Chinese Center for Disease Control and Prevention, Beijing, China; 4 Department of Epidemiology, IUF-Leibniz Research Institute for Environmental Medicine, Düsseldorf, Germany; 5 Department of Big Data in Health Science, School of Public Health, Zhejiang University, Hangzhou, China; 6 Faculty of Medicine, University of São Paulo, São Paulo, Brazil; 7 Taierzhuang Center for Disease Control and Prevention, Zaozhuang, China; 8 Management and Policy, School of Public Health, Cheeloo College of Medicine, Shandong University, Jinan, China; 9 National Health Commission of China Key Laboratory of Health Economics and Policy Research, Shandong University, Jinan, China

**Keywords:** influenza, cold spell, definition, vulnerable population, modification effect, China

## Abstract

**Background:**

Studies have reported the adverse effects of cold events on influenza. However, the role of critical factors, such as characteristics of cold spells, and regional variations remain unresolved.

**Objective:**

We aimed to systematically evaluate the association between cold spells and influenza incidence in mainland China.

**Methods:**

This time series analysis used surveillance data of daily influenza from 325 sites in China in the 2014‐2019 period. A total of 15 definitions of cold spells were adopted based on combinations of temperature thresholds and days of duration. A distributed lag linear model was used to estimate the short-term effects of cold spells on influenza incidence during the cool seasons (November to March), and we further explored the potential impact of cold spell characteristics (ie, intensity, duration, and timing during the season) on the estimated associations. Meta-regressions were used to evaluate the modification effect of city-level socioeconomic indicators.

**Results:**

The overall effect of cold spells on influenza incidence increased with the temperature threshold used to define cold spells, whereas the added effects were generally small and not statistically significant. The relative risk of influenza-associated with cold spells was 3.35 (95% CI 2.89‐3.88), and the estimated effects were stronger during the middle period of cool seasons. The health effects of cold spells varied geographically and residents in Jiangnan region were vulnerable groups (relative risk 7.36, 95% CI 5.44‐9.95). The overall effects of cold spells were positively correlated with the urban population density, population size, gross domestic product per capita, and urbanization rate, indicating a sterner response to cold spells in metropolises.

**Conclusions:**

Cold spells create a substantial health burden on seasonal influenza in China. Findings on regional and socioeconomic differences in the health effects of cold spells on seasonal influenza may be useful in formulating region-specific public health policies to address the hazardous effects of cold spells.

## Introduction

Seasonal influenza causes substantial morbidity and mortality worldwide each year, with an estimated 291,000 to 645,000 seasonal influenza-related deaths annually [1]. The disease and economic burdens of seasonal influenza are substantial in China. It is estimated that an average of 88,100 influenza-related excess respiratory deaths occur each year in mainland China [2]. The influence of ambient low temperature on influenza has been well documented. Climate change is a major threat in the 21st century that may pervasively affect human life by increasing the frequency and intensity of extreme temperature events [3]. Numerous studies have confirmed that nonoptimum temperatures are associated with a higher risk of morbidity and mortality, with most related disease burdens being more explainable by cold exposure than by heat exposure [4-7]. There has been sound evidence suggesting that human body’s adaptation to heat is more efficient than its adaptation to cold in the context of global warming [8]. However, relatively limited attention has been paid to the health effects of cold weather, despite its greater impact and weaker adaptability of individuals to it [9]. Therefore, the health risks of seasonal influenza related to the cold should not be ignored.

Seasonal influenza is often characterized by a sharp peak during winter months in temperate regions. Low temperature conditions during the cool seasons may increase the stability of influenza virus particles remaining on the mucosa of the upper respiratory tract and promote the transmission of the influenza virus [10]. High-intensity cold spells, also known as Siberian cold currents, occur frequently during the cool seasons in China, which might be involved in the epidemics of seasonal influenza [11]. However, evidence evaluating the impact of cold spells on seasonal influenza in China is limited. To date, the definition of cold spell is inconclusive around the world. The most commonly accepted definition of cold spell is an extreme cold weather event that lasts for several days [11]. Previous studies have suggested the adverse effects of cold spells can be decomposed into two parts: overall and added effects [12]. The overall effect is the increased risk due to exposure to daily low temperatures, while the added effect is the additional risk from the sustained duration of low temperatures [13]. However, some studies have reported the added effects of extreme temperatures, while others suggested that the added effects of extreme temperatures were negligible [14]. Therefore, the effect of cold spells on public health is unclear. Moreover, a few studies revealed that the health effects of extreme temperatures are associated with their characteristics, such as intensity, duration, and timing, during the season; however, little is currently known about the impact of these characteristics of cold spells on influenza [15].

Studies have found that associations between cold and health vary substantially by location [12]. Given the vast geographic area and climatic diversity of China, a large-scale multicity study to evaluate the impact of cold spells on influenza in different climatic zones is meaningful and necessary to support region-specific intervention strategies. At the population level, the adverse effects of cold are not evenly distributed [11]. For example, a study conducted in China indicated that people with lower socioeconomic status tend to be more vulnerable to cold-related mortality [16]. However, whether similar socioeconomic inequalities exist in susceptibility to influenza during cold spells remains largely unknown. Further investigation is needed to better understand the increased risk of seasonal influenza-related to cold spells.

In this study, we quantified the association between cold spells and influenza incidence across mainland China from 2014 to 2019. Specifically, we used a national data set to examine (1) the variation in the strength of this association under 15 different cold spell definitions; (2) whether cold spells had added effects on influenza incidence; (3) the potential influence of cold spell characteristics (ie, intensity, duration, and seasonal timing); and (4) the estimated effects of cold spells in different climatic zones and socioeconomic levels. The findings were expected to formulate tailored preventive policies for seasonal influenza and to address the complex challenges posed by extreme climate change in China.

## Methods

### Study Area

Mainland China has 31 provincial divisions (including 4 municipalities), 333 prefectures-level divisions, and 2853 county-level divisions (including 27 counties under provincial jurisdiction by the end of 2013) [17]. In this study, the unit of data collection and analysis was confined to the city level, including four municipalities, 333 prefectural-level divisions, and 27 counties under provincial jurisdiction. To reduce modeling instability introduced by limited influenza cases in small cities, an inclusion criterion of above 10% of total influenza cases from 2014 to 2019 was adopted. Finally, 325 cities were enrolled in the formal analysis, which was further divided into 8 meteorological-geographic regions [18] according to the China meteorology administration (Figure S1 in Multimedia Appendix 1).

### Data Sources

Data on daily seasonal influenza surveillance from January 1, 2014, to December 31, 2019, were obtained from the National Center for Disease Control and Prevention of China. Influenza is a class C notifiable infectious disease in China, and all cases must be reported electronically within 24 hours according to the National Communicable Disease Control Act. The diagnosis was based on the standard diagnostic criteria for influenza (WS 285‐2008) issued by the National Health Commission of the People’s Republic of China [19].

Daily meteorological data during the study period, including mean temperature, relative humidity, cumulative precipitation, wind speed, and sunshine hours, were collected from the China Meteorological Data Sharing Service System. The missing data on a certain day were imputed using the average values of the adjacent 2 days. We collected the city-level socioeconomic characteristics from the China City Statistical Yearbook, including demographic variables (eg, population number [persons], population density [person/km^2^], population growth rate [%], and urbanization rate [%]), economic variables (gross domestic product [GDP] per capita, in Chinese Yuan), health resources (hospital beds per 1000 persons), air pollution (average annual concentration of PM_2.5_ [μg/m^3^]), and collections of public libraries per 100 persons.

### Statistical Analysis

There is no unified definition for cold spells worldwide, although they are commonly defined as a few consecutive days with low temperatures below a specific threshold during the cool seasons [11]. In this study, we used 15 city-specific definitions of cold spells by combining 5 temperature thresholds (mean temperature under the 1st, 2nd, 3rd, 4th, and 5th percentiles of the city-specific daily mean temperature distribution) and 3 durations (at least 2, 3, or 4 consecutive days with daily mean temperature below the thresholds). Considering that most cold spells occurred in cold months of the year, data analyses were limited to the cool season (November to March). We applied a 2-stage analysis to estimate the association between cold spells and influenza incidence. First, a quasi-Poisson regression with distributed lag model (DLM) was used to evaluate the association for each city [20]. DLM can simultaneously control the conventional exposure-response relationship and the additional delayed effect of exposure factor through a cross-basis function [11,20]. The model was expressed as follows (Equation 1):


(1) $$Log(\mu_{t})=\alpha +\beta T_{t,l}(CSt)+\;ns(mete,3)+ns(time,4\;per\;year)+dow$$

where μ*_t_* is the estimated number of influenza cases on day *t*; *α* is the intercept; *CS_t_* is a binary variable for a cold spell day; *T_t,l_* (.) means cross-basis functions obtained by applying the DLM to *CS_t_* for the overall effects of cold spells, with a line function for exposure-response dimension and a natural cubic spline function with 5 df for lag-response dimension [12]. A maximum lag of 21 days was selected to account for delayed effects; “*ns*(mete,3)” represents the natural spline function of relative humidity and cumulative precipitation [14]. A natural cubic spline of calendar time with 4 df per year was used to control for seasonality and long-term trends in influenza incidence; “dow” is a categorical variable representing the day of the week. In the second stage, city-specific influenza incidence risks were pooled at national and regional levels using random-effect meta-analyses with maximum likelihood estimation [21]. Akaike information criterion for quasi-Poisson regression was used to evaluate the goodness of model fits of 15 cold spell definitions. In addition, we conducted stratification analyses in different climatic zones of China under the optimal definition of cold spells. The statistical significance of differences in the risk estimates between different climatic zones was tested by calculating the 95% CIs with the following formula:


$$(\hat{Q_{1}}-\hat{Q_{2}})\pm 1.96\sqrt{{\hat{SE_{1}}}^{2}+{\hat{SE_{2}}}^{2}}$$

where $\hat{Q_{1}}$ and $\hat{Q_{2}}$ are the estimated effect values of 2 subgroups, and $\hat{SE_{1}}$ and $\hat{SE_{2}}$ are their respective standard errors [11]. To examine whether cold spells had added effects on influenza incidence, beyond the effect of single days of cold temperature, the added effect of cold spells on influenza was estimated by controlling for ambient temperature effects in the above first-stage model [12]. Here, we used a cross-basis function with a 3-df natural cubic spline for temperature and a 5-df natural cubic spline for lag of temperature up to 21 days to control the temperature effects. Then, the random-effect meta-analysis was performed to pool city-specific added effects of cold spells at national and regional levels.

To estimate the effects of the intensity, duration, and seasonal timing of cold spells on influenza incidence, we replaced the model 1 (ie, Equation 1) with the following model (ie, Equation 2):


(2) $$log(\mu_{t})=\alpha +\beta_{1}CI_{t,l}+\beta_{2}CD_{t,l}+\;\beta_{3}CT_{t,l}+ns(mete,3)+ns(time,4\;per\;year)+dow$$

where $CI_{t,l}$ represents the intensity of cold spells on day *t*, defined as the difference between daily mean temperature and the city-specific threshold, which is 0 when temperatures are at or above the threshold; $CD_{t,l}$ is a continuous variable indicating the duration of cold spells, which is 0 on the first day of a cold spell, 1 on the second day, and so on; $CT_{t,l}$ denotes the seasonal timing of cold spells, defined as the difference in days between day *t* and the first day (ie, November 1) of the cool seasons, which is is 0 on days without cold spells [15]. Specifically, we estimated the impacts of each cold-spell characteristic in each city using quadratic splines without natural constraints and 2 equally spaced knots and then pooled using random effect meta-analysis [13,21].

The Spearman rank test was used to explore the correlation between socioeconomic factors and the overall effects of cold spells on influenza incidence at the national level. Then, the pooled estimation of the association between cold spells and influenza incidence for cities in different socioeconomic groups (quartiles 1-4 of each socioeconomic indicator) was obtained using a random-effect meta-analysis with maximum likelihood estimation. We quantified socioeconomic inequality in influenza vulnerability associated with cold spells, and the difference in relative risks (RR) between different socioeconomic subgroups was estimated by the meta-regression [12].

To test the robustness of our results, we performed sensitivity analyses by changing the location of knots for cold spells, the maximum lag days, and the df for seasonality and long-term trends in model 1 and model 2, respectively. we also tested whether the socioeconomic disparities in residents’ vulnerability to influenza during cold spells remained significant after adjusting for potential effects of population structure (the proportion of children aged <5 years and the proportion of older adults aged >60 years) in the meta-regression model. All statistics were performed using R (version 3.6.3; R Fouondation for Statistical Computing), and a 2-sided *P*<.05 was set as statistically significant for all statistical tests [12].

### Ethical Considerations

This study was approved by the Ethics Review Committee, School of Public Health, Shandong University (20221116), and informed consent was waived because all the data were deidentified and aggregated at the city level.

## Results

### Data Description

A total of 4,006,056 influenza cases were notified over the study period in mainland China, 99.88% (4,001,355) of which occurred in the target 325 cities (Figure S2 in Multimedia Appendix 1). Most reported influenza cases occurred in the eastern part of China, whereas the western and northeastern parts of China are sparsely populated and have relatively few reported influenza cases. An inverted V-shaped curve was observed between temperature and influenza incidence during the cool seasons at the national level, indicating ambient low temperatures were associated with increased morbidity risk (Figure S3 in Multimedia Appendix 1).

### Effects of Cold Spells Under Different Definitions

**Figure 1.**
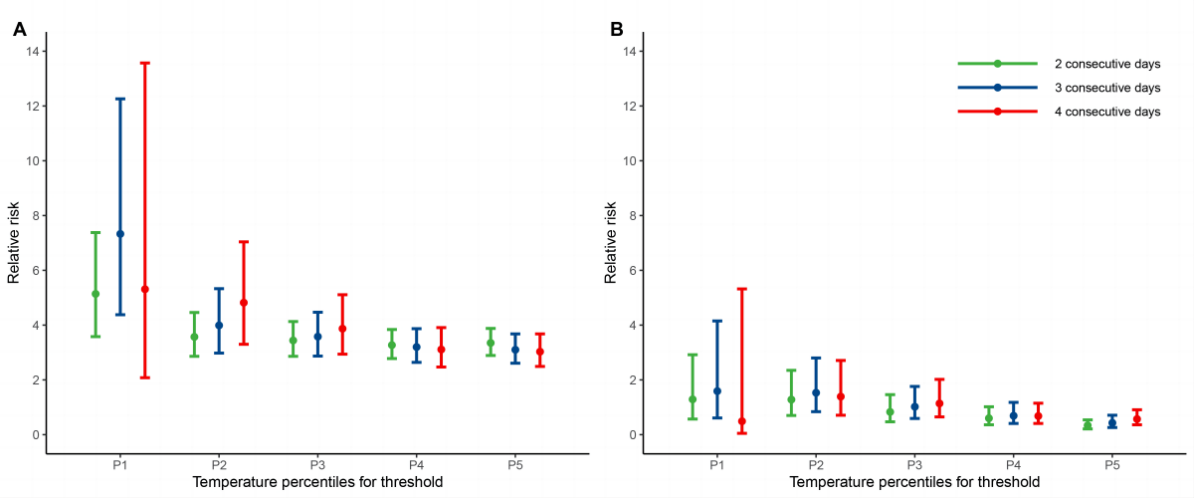
Relative risk of influenza incidence due to the (A) overall and (B) added effects of cold spells. P1, P2, P3, P4, and P5 denote the first, second, third, fourth, and fifth percentiles of the temperature distribution, respectively.

**Figure 2.**
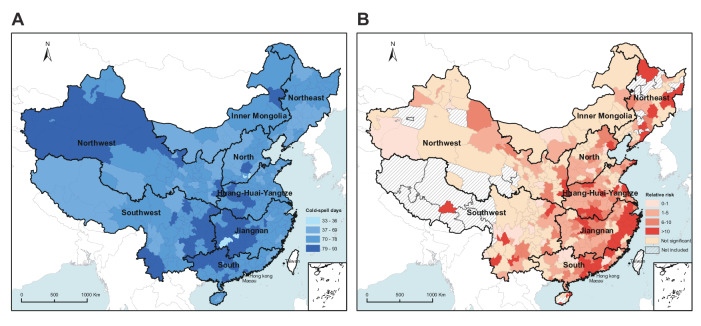
Distribution of cold spells and the overall effects of cold spells on seasonal influenza.

In general, the lower the temperature threshold used to define cold spells, the higher the cold spell associations with influenza incidence (Figure 1). We identified 1274 cold spell days in 315 cities under the strictest cold spell definition (temperature below the 1st percentile, lasting 4 days), and an approximately 19-times increase in cold spell days (23,994 days) was noted for the mildest cold-spell definition (temperature below the 5th percentile, lasting 2 days). Compared with the overall effects of cold spells, the added effects were small and mostly not statistically significant (Table S1 in Multimedia Appendix 1). We found considerable heterogeneity among the overall and added estimates across cities (all heterogeneity: *P*<.001). For overall cold spell effects, our results showed that cold spells defined as daily mean temperature falling below the city-specific 5th temperature percentile for at least 2 consecutive days produced the optimum model fit performance (Figure S4 in Multimedia Appendix 1). Figure 2A shows the geographical distribution of cold spells under the optimum cold spell definition. Cold spells were more common in the western part of the northwestern region and the border area between the southwest and Jiangnan regions. The RR of seasonal influenza associated with cold spells varied greatly by city and region, with an increased risk of infection most apparent in the coastal area of Jiangnan and southern China (Figure 2B). For climatic zones, the overall effects of cold spells varied greatly by climatic region, and people in the Jiangnan region were more vulnerable to seasonal influenza during cold spells (Table S2 in Multimedia Appendix 1).

### Effects of Cold Spell Characteristics

**Figure 3.**
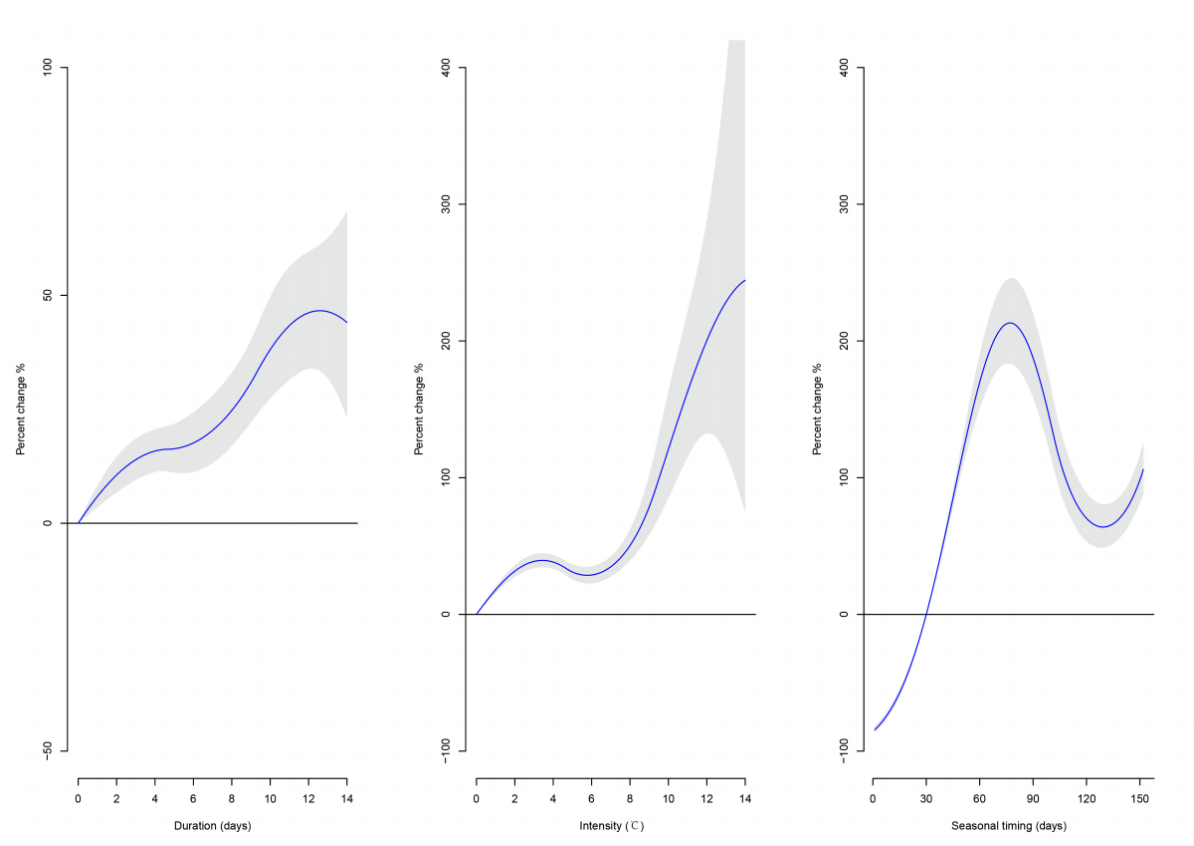
Percent changes in influenza incidence associated with cold spell characteristics.

The pooled percentage increase in influenza incidence associated with cold spell characteristics in China is shown in Figure 3. At the national level, there was an approximately positive linear association between influenza incidence and the duration of cold spells, with the number of influenza cases increasing by 16.3% (95% CI 11.1%‐21.8%) for each 5-day increase in cold spell duration. The results suggest an N-shaped curve between influenza incidence and cold spell intensity; each 5 ℃ increase in cold spell intensity leads to a 31.0% (95% CI 25.7%‐36.5%) increase in the number of influenza cases. A higher risk of influenza associated with cold spells occurred in the middle of the cool season, and there was an approximately linear increase trend in influenza risk early in the cool season. We also observed an upward trend for the effects of timing later in the cool season (February to March). Stratified analysis illustrated that the effect of cold spell characteristics showed spatial heterogeneity across various climate zones (Table S3 in Multimedia Appendix 1).

### Modifying Effects of Socioeconomic Indicators

**Figure 4.**
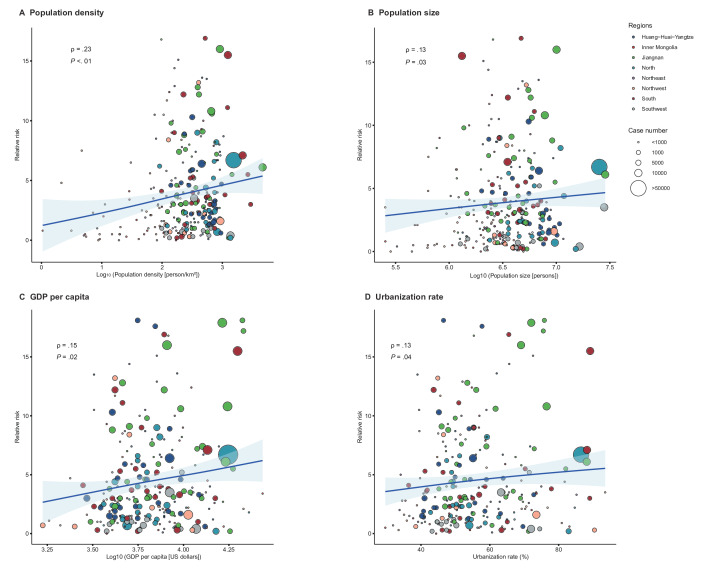
The correlation between the overall effects of cold spells and socioeconomic factors. GDP: gross domestic product.

**Figure 5.**
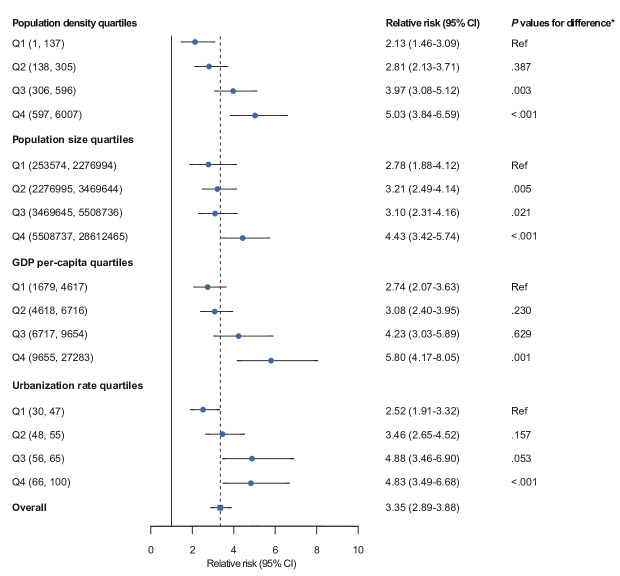
Overall effects of cold spells on seasonal influenza incidence, stratified by socioeconomic level. GDP: gross domestic product.

The overall effects of cold spells on seasonal influenza were positively correlated with urban population density, population size, GDP per capita, and urbanization rate (Figure 4 and S4 in Multimedia Appendix 1). At the national level, the RR of seasonal influenza associated with cold spells was 3.35 (95% CI 2.89‐3.88). The association between cold spells and seasonal influenza was stronger in cities with higher population density, population size, GDP per capita, and urbanization rates than in cities with lower levels of socioeconomic indicators (Figure 5). Specifically, the RR associated with cold spells was 5.03 (95% CI 3.84‐6.59) for cities with high population density (597‐6007 person/km^2^), 4.43 (95% CI 3.42‐5.74) for cities with large populations (5,508,737‐28,612,465 persons), 5.80 (95% CI 4.17‐8.05) for high-income cities (GDP per capita: 69,001‐195,000 Chinese Yuan, which equals about US $9654 to US $27,283), and 4.83 (95% CI 3.49‐6.68) for highly urbanized cities (urbanization rate: 66%‐100%). No significant associations were detected between the overall effects of cold spells and population growth rate, hospital beds, collections of public libraries, and annual average PM_2.5_ concentrations (Figure S5 in Multimedia Appendix 1).

The effect estimates of the overall and added effects of cold spells and the percentage increase in influenza incidence associated with cold spell characteristics were broadly similar when we conducted sensitivity analyses (Table S5 and Figure S6 in Multimedia Appendix 1). After adjusting for city-level population structure in the meta-regression, the city-specific RRs still showed significant positive associations with the population density, population size, GDP per capita, and urbanization rates (Table S6 in Multimedia Appendix 1). These results suggested that our main results were robust.

## Discussion

### Principal Findings

This study examined the impact of cold spells on influenza incidence under various cold spell definitions in mainland China during cool seasons, investigated how the risk of influenza depended on cold spell characteristics, and assessed whether the association between cold spells and influenza incidence was affected by local socioeconomic level. Results indicate that the adverse effect of cold spells on influenza incidence increased with the temperature threshold in the cold spell definition, with the estimated effects of cold spells being stronger in the middle period of the cold season. Population density, population size, per capita income, and urbanization rate were found to be positively correlated with the morbidity risk of influenza caused by cold spells.

We quantified how much of the excess risk of influenza during cold spells was explained by the independent effects of low temperatures (overall effects of cold spells) and how much was caused by consecutive days of cold temperatures (added effects of cold spells). The results showed that most of the excess risk of influenza during cold spells can be attributed to the decrease of daily mean temperature, which was similar to the findings of previous studies. For example, a study conducted in China revealed that the association between cold spells and mortality was largely attributable to extreme low temperatures rather than sustained cold days [22]. The study by Song et al [14] found that the excess risk of respiratory emergency department visits during cold spells in Beijing, China, was mainly due to the drop in daily temperatures. Compared with the overall effect of cold spells, the added effect of cold spells was small and not statistically significant among most cold spell definitions. This was consistent with the previous findings by Lin et al [23]. Furthermore, the observed overall effects of cold spells on influenza incidence in this study varied with the cold spell definition we used. The adverse effects of cold spells were noted even under the mildest definition of cold spells, suggesting that reducing the harmful effects of cold spells on seasonal influenza should be a significant public concern.

Various potential mechanisms have been postulated to account for the higher morbidity of influenza during cold spells. Inhalation of cold air could cool the nasal epithelium and impair the phagocytic activity of leukocyte and mucociliary clearance, thus inhibiting respiratory defenses against viral infection [24]. An animal experiment on guinea pigs found that cold and dry meteorological conditions could facilitate the transmission of influenza viruses [25]. Another mechanism suggests that a decrease in temperature could increase the shedding of influenza viruses, making airways more susceptible to respiratory infections and increasing host’s susceptibility [26,27]. In addition, cold spells may drive people to move indoors, which may increase the risk of cross-infection with influenza from indoor crowding [19].

Our analysis indicated that cold spells were more prevalent in the western part of the northwestern region and the border area between the southwest and Jiangnan regions. Similar to our findings, the study by sun et al [28] revealed that the frequency of cold spells was higher in the northwest and central China. Located in the northwest of China, Xinjiang has a fragile ecosystem with limited adaptation to climatic conditions and is highly vulnerable to extreme weather events [29]. The higher frequency of cold spells in central China may be due to the higher temperature thresholds for cold spells in those areas [28]. Therefore, the frequency of cold spell occurrences during cold seasons in these two regions was high. On the other hand, spatial variation was observed in the effect estimates of cold spells on influenza incidence among various climatic zones. Our study demonstrated that the overall effects of cold spells was significantly higher in Jiangnan region, indicating that people in Jiangnan region were more susceptible to temperature drops during cold spells. In line with our results, previous studies have shown that the effects of cold spells are greater in the southern part of China, the United States, and European countries [30-32]. People living in cold climates tend to be more accustomed to and prepared for extreme cold temperature than those in warmer climates [30]. Another possible reason is the geographical differences in adaptation measures to cold weather in homes and communities, with few buildings equipped with heating systems in southern China [30,33]. The regional disparities in the occurrence of cold spells and the risk of seasonal influenza emphasized the geographic variability and complexity of climatic influences on seasonal influenza activity and warrant further investigation.

We observed an approximately linear increase in the effect of cold spells on influenza incidence as the duration of cold spells increased. This finding is in accordance with some previous findings. For example, a study exploring mortality and cold spells in 66 Chinese communities during 2006 to 2011 found that the excess risk of nonaccidental mortality was 28.2% and 58.6% for cold spells lasting for 2 and ≥6 days, respectively [33]. Liu et al [8] found that the effects of cold spells on childhood asthma were not constant from day to day, and the effect of cold spell increased with its duration. This result indicated that more comprehensive protection measures are needed to reduce the excessive risk of cold spells. We also found that the association between cold spells and influenza incidence is generally stronger for cold spells that are more intense. Sudden temperature changes may affect humoral and cellular immunity, increase respiratory workload and induce the occurrence of respiratory infections [34]. Another plausible explanation is that stronger cold temperatures could promote indoor activities and thus enhance contact transmission of influenza viruses [19]. In addition, our study illustrates that there was a remarkably upward trend in the risk of influenza during the early period of cool seasons, which is in line with previous studies [33,35]. Preparations for cold weather, including home heating fuel and warm clothing, may be inadequate early in the cool seasons [15]. Another explanation is that the body needs time to adapt to changes in ambient temperature, and if the temperature drops sharply and suddenly earlier in the cool seasons, the body may not respond in time [33]. The finding highlights the importance of strengthening preventive measures for influenza virus infections early in the cool seasons. We also found stronger associations between influenza incidence and cold spells that occurred later in the cool seasons, which may be related to the increased activity of influenza viruses [36].

In this nationwide study, we showed that population density, population size, and urbanization rate were positively correlated with the morbidity risk of influenza caused by cold spells, suggesting a higher transmission risk of influenza in metropolises during cold spell days. Consistent with our finding, previous studies have indicated that cities differ in population size and spatial structure may affect infectious transmission patterns. The potential for influenza transmission in metropolises may be elevated due to the clustering of buildings, such as homes and workplaces, and the prevalence of high-density public transportation [37]. Moreover, variation in urbanization rates may also cause divergent epidemic dynamics of seasonal influenza at the city level. For example, Lei et al [38] reported a U-shaped relationship between influenza attack rates and urbanization rate in China, with a nearly linear increase in attack rates when the urbanization rate is above 60%. The acceleration of urbanization with no relevant interventions has the potential to worsen epidemiological dynamics of influenza [39]. It is worth noting that we found the association between cold spells and seasonal influenza was stronger in high-income cities. One possible explanation is that an increasing number of rural migrant workers have moved and continue to move to large cities for job opportunities, while their living standard, sanitation, and health care levels are relatively poor [40]. Additionally, older individuals are vulnerable to temperature variability due to their decreased thermoregulatory functioning and weaker immune systems [41]. China has experienced rapid population aging in recent decades; the changing demographic profiles of large cities due to the continued population aging may be another important reason. However, previous studies suggested that persons with a lower socioeconomic status were at higher risk for hospitalization with influenza due to the crowded conditions within the home and community, and lack of health-care services [42,43]. This seemingly contradictory result reflects the socioeconomic differences and complexity of the impact of climate extremes on influenza activity. Controlling influenza epidemics is an important public health goal, and our findings highlight the potential challenges of cold spells on population health in metropolises.

### Study Limitations

There were some limitations to this study. First, meteorological data from fixed-site monitoring stations might not represent personal exposure, which may lead to exposure measurement bias. Second, our study period was only 6 years and the statistical power of this study could be attenuated, but this may not substantially affect our findings at the national level [11]. Third, our research was considered an ecological study, and risk factors at the individual level, such as host behavior and susceptibility, which may impact the risk of influenza virtual infection, are not considered. Finally, we did not further analyze the influence of cold spells on different types of seasonal influenza viruses due to the lack of data on the pathogen classification of influenza viruses.

### Conclusions

Cold spells significantly increased the morbidity of seasonal influenza in China, especially in the Jiangnan region and in metropolitan areas, and the effect varied with climatic zones and socioeconomic levels. The influence of cold spells is closely related to their intensity, duration, and seasonal timing. Cold spells remain a serious health problem in China, and our findings have significant implications for developing precise influenza prediction and prevention measures during cool seasons.

## Supplementary material

10.2196/55822 Multimedia Appendix 1 Additional statistics.

## Abbreviations

DLM: distributed lag mode
GDP: gross domestic product
RR: relative risk
