# Supplementary material for: Increased Risk of Influenza Infection During Cold Spells in China: National Time Series Study
Source: JMIR Public Health Surveill. 2024 Aug 13;10:e55822. doi: 10.2196/55822 (PMC11336504; doi:10.2196/55822)
Supplement: Multimedia Appendix 1 [file publichealth-v10-e55822-s001.docx]

**Supplemental material**

**Fig. S1** Geographic locations and mean temperatures in cool seasons for the 325 cities.

**Fig. S2** Geographic distribution of influenza cases in cool seasons from 2014 to 2019 in 325 cities of China.

**Fig. S3** Associations between ambient temperatures and influenza during the study period.

**Fig. S4** The overall model fits from the 325 cities under 15 definition cold spells.

**Table S1.** Pooled overall and added effects of cold spells on influenza incidence under 15 different cold spells definitions.

**Table S2.** Pooled overall and added effects of cold spells on influenza incidence under the optimum cold spell definition in different regions.

**Table S3.** Percentage change (95%CI) in influenza incidence with per unit increase in cold spell characteristic during cool seasons in China, 2014–2019.

**Table S4.** Spearman’s correlation coefficients between different sociodemographic factors.

**Fig. S5** The correlation between some sociodemographic factors and the relative risk of influenza caused by cold spells.

**Table S5.** Sensitivity analysis by changing the degrees of freedom (df) for temperature, maximum lag days, long-term trends and seasonality on the pooled overall and added effects under the optimum cold spell definition.

**Fig. S6** Sensitivity analysis by changing the df for temperature (A) and long-term trends and seasonality (B) on the percent changes in influenza incidence associated with the cold spells characteristics.

**Table S6.** Relationship between city-specific socioeconomic indicators and overall estimates of the association between cold spells and influenza incidence.

**Fig. S1** Geographic locations and mean temperatures in cool seasons for the 325 cities.


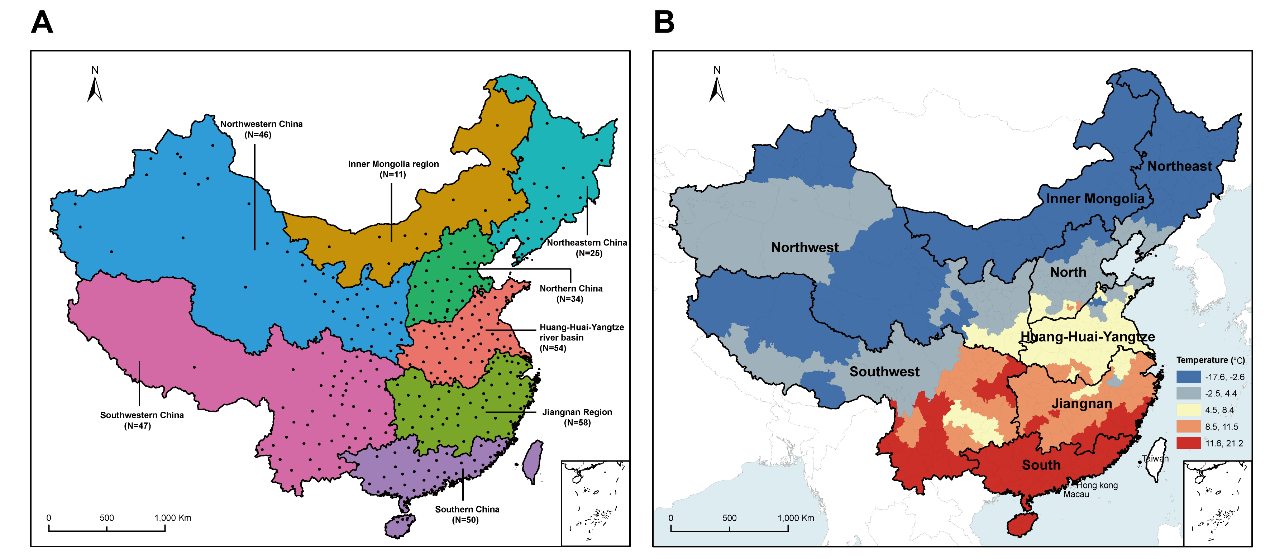


**Fig. S2** Geographic distribution of influenza cases in cool seasons from 2014 to 2019 in 325 cities of China.


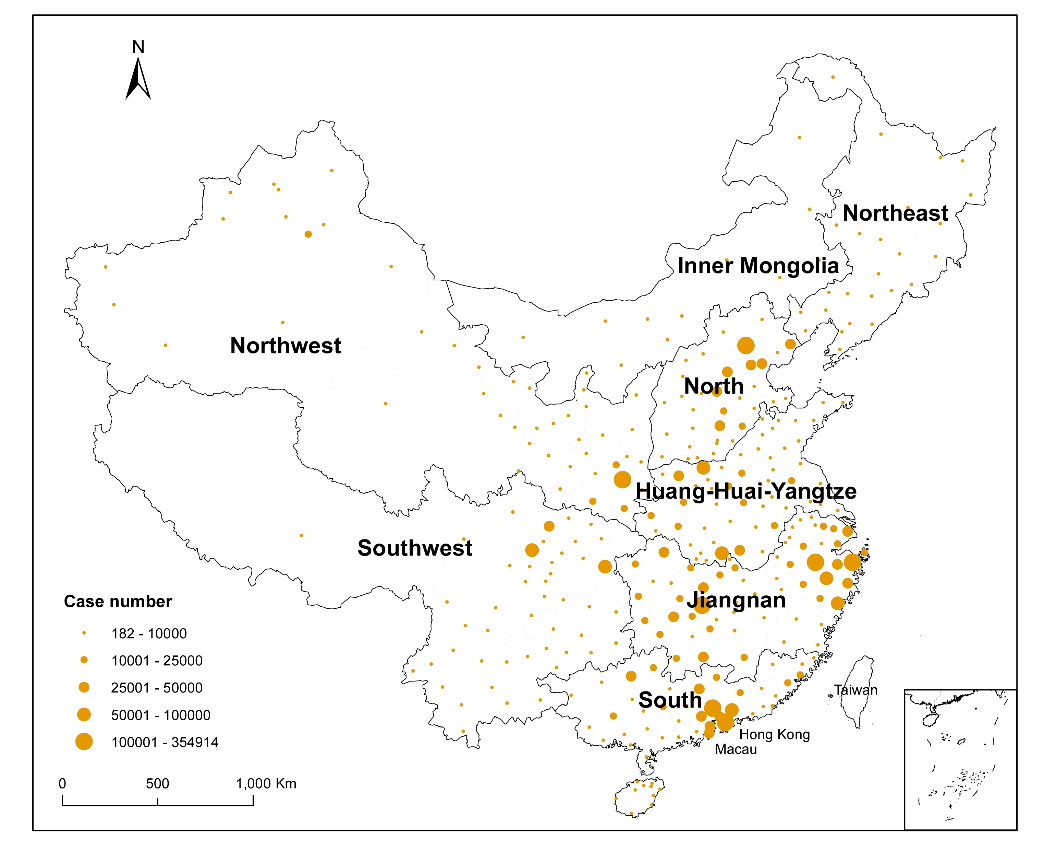


**Fig. S3** Associations between ambient temperatures and influenza during the study period.


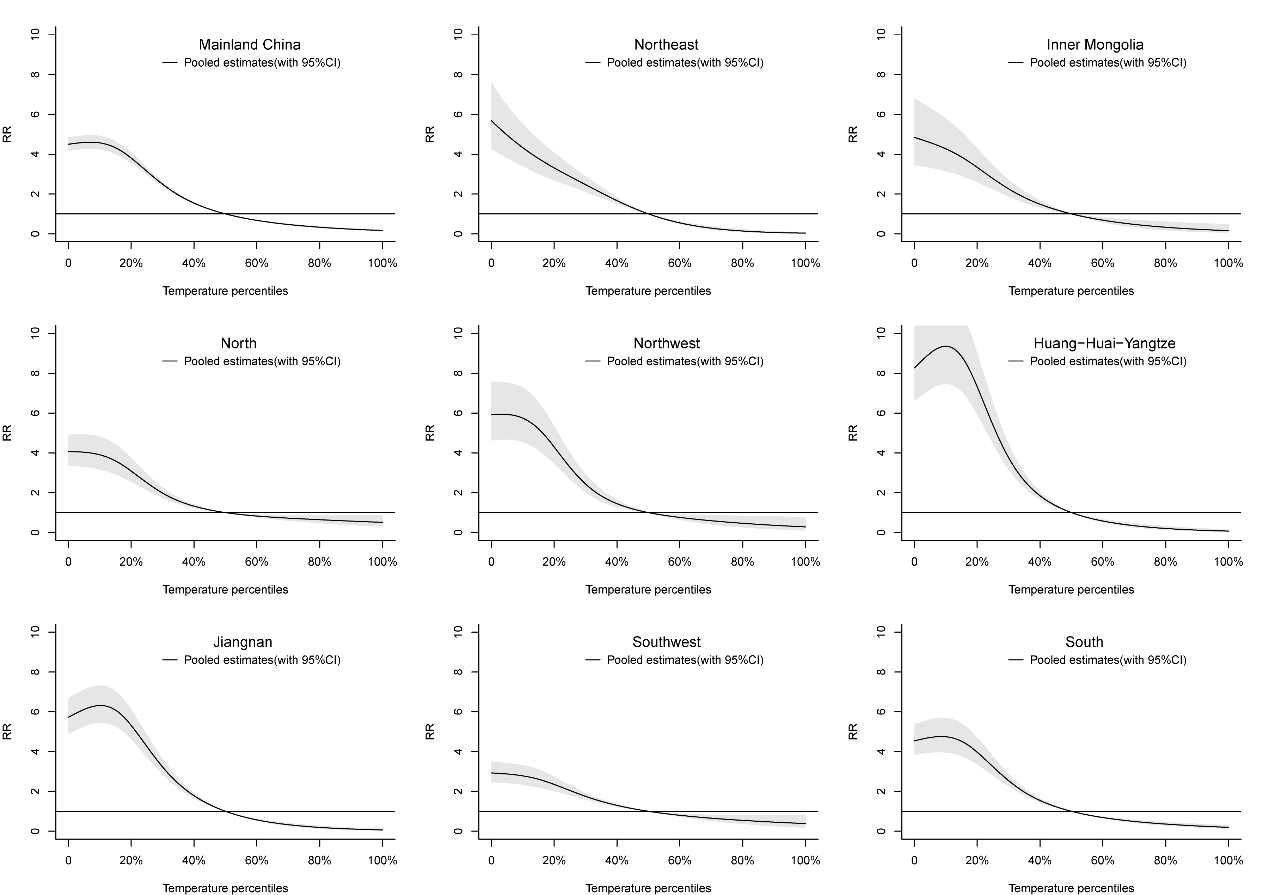


**Fig. S4** The overall model fits from the 325 cities under 15 definition cold spells.

**
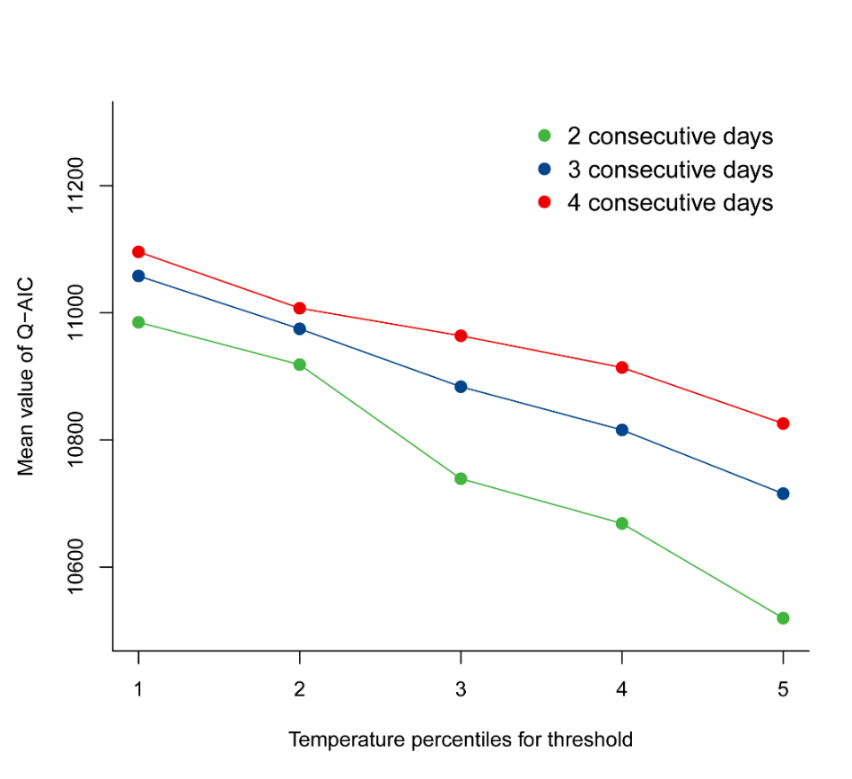
**

**Table S1.** Pooled overall and added effects of cold spells on influenza incidence under 15 different cold spells definitions.

| **Number**  **of days** | **Temperature**  **percentiles** |  | **Number**  **of cities** |  | **Total number of**  **cold spell days** |  | **Overall effects** | |  | **Added effects** | |
| --- | --- | --- | --- | --- | --- | --- | --- | --- | --- | --- | --- |
|  |  |  |  |  |  |  | **RR (95%CI)** | **Test for Heterogeneity** |  | **RR (95%CI)** | **Test for Heterogeneity** |
| ≥2 | ≤1st |  | 325 |  | 4072 |  | 5.14 (3.58-7.38) | <.001 |  | 1.29 (0.57-2.92) | <.001 |
|  | ≤2nd |  | 325 |  | 8702 |  | 3.57 (2.86-4.46) | <.001 |  | 1.28 (0.70-2.35) | <.001 |
|  | ≤3rd |  | 325 |  | 13620 |  | 3.44 (2.86-4.13) | <.001 |  | 0.83 (0.47-1.46) | <.001 |
|  | ≤4th |  | 325 |  | 18672 |  | 3.27 (2.78-3.84) | <.001 |  | 0.60 (0.36-1.02) | <.001 |
|  | ≤5th |  | 325 |  | 23994 |  | 3.35 (2.89-3.88) | <.001 |  | 0.34 (0.21-0.54) | <.001 |
| ≥3 | ≤1st |  | 325 |  | 2286 |  | 7.33 (4.38-12.26) | <.001 |  | 1.59 (0.61-4.15) | <.001 |
|  | ≤2nd |  | 325 |  | 5341 |  | 3.99 (2.98-5.33) | <.001 |  | 1.53 (0.84-2.80) | <.001 |
|  | ≤3rd |  | 325 |  | 8681 |  | 3.58 (2.87-4.47) | <.001 |  | 1.02 (0.59-1.76) | <.001 |
|  | ≤4th |  | 325 |  | 12356 |  | 3.20 (2.64-3.87) | <.001 |  | 0.69 (0.41-1.18) | <.001 |
|  | ≤5th |  | 325 |  | 16341 |  | 3.10 (2.61-3.68) | <.001 |  | 0.43 (0.26-0.71) | <.001 |
| ≥4 | ≤1st |  | 315 |  | 1274 |  | 5.31 (2.08-13.57) | <.001 |  | 0.49 (0.05-5.32) | <.001 |
|  | ≤2nd |  | 325 |  | 3386 |  | 4.82 (3.30-7.04) | <.001 |  | 1.39 (0.71-2.71) | <.001 |
|  | ≤3rd |  | 325 |  | 5705 |  | 3.87 (2.94-5.11) | <.001 |  | 1.14 (0.65-2.02) | <.001 |
|  | ≤4th |  | 325 |  | 8421 |  | 3.11 (2.47-3.91) | <.001 |  | 0.68 (0.41-1.15) | <.001 |
|  | ≤5th |  | 325 |  | 11578 |  | 3.03 (2.49-3.68) | <.001 |  | 0.57 (0.36-0.91) | <.001 |

**Table S2.** Pooled overall and added effects of cold spells on influenza incidence under the optimum cold spell definition in different regions.

| **Regions** | **Overall effects** | | | **Difference test** |  | **Added effects** | | |  |
| --- | --- | --- | --- | --- | --- | --- | --- | --- | --- |
|  | **RR (95%CI)** | ***β*** | ***Se*** |  |  | **RR (95%CI)** | ***β*** | ***Se*** | **Difference test** |
| Northeast | 4.55 (2.36, 8.80) | 1.52 | 0.34 | (-0.37, 0.99) |  | 0.70 (0.14, 3.50) | -0.36 | 0.82 | (-1.45, 1.85) |
| Inner Mongolia | 1.23 (0.71, 2.10) | 0.20 | 0.28 | (-1.58, -0.44)^a^ |  | 0.20 (0.05, 0.79) | -1.63 | 0.71 | (-2.52, 0.38) |
| Northwest | 1.45 (1.06, 1.99) | 0.37 | 0.16 | (-1.19, -0.49)^a^ |  | 0.63 (0.24, 1.65) | -0.46 | 0.49 | (-0.94, 1.14) |
| North | 2.52 (1.87, 3.41) | 0.93 | 0.15 | (-0.61, 0.05) |  | 0.20 (0.09, 0.46) | -1.62 | 0.43 | (-1.99, -0.13)^a^ |
| Huang-Huai-Yangtze | 4.39 (3.27, 5.89) | 1.48 | 0.15 | (-0.06, 0.60) |  | 0.36 (0.16, 0.80) | -1.02 | 0.41 | (-1.35, 0.43) |
| Jiangnan | 7.36 (5.44, 9.95) | 2.00 | 0.15 | (0.46, 1.12)^a^ |  | 0.62 (0.27, 1.43) | -0.47 | 0.42 | (-0.82, 1.00) |
| Southwest | 2.09 (1.38, 3.16) | 0.74 | 0.21 | (-0.91, -0.03)^a^ |  | 0.34 (0.12, 0.96) | -1.09 | 0.53 | (-1.64, 0.58) |
| South | 4.29 (2.92, 6.30) | 1.46 | 0.20 | (-0.17, 0.67) |  | 3.36 (1.01, 11.15) | 1.21 | 0.61 | (0.51, 3.03)^a^ |
| China mainland | 3.35 (2.89, 3.88) | 1.21 | 0.08 | Ref |  | 0.57 (0.39, 0.84) | -0.56 | 0.20 | Ref |

^a^*P*<.05.

**Table S3.** Percentage change (95% CI) in influenza incidence with per unit increase in cold spell characteristic during cool seasons in China, 2014–2019.

| **Regions** | **Duration** | | |  | **Intensity** | | |  | **Seasonal timing** | | |
| --- | --- | --- | --- | --- | --- | --- | --- | --- | --- | --- | --- |
|  | **2 day** | **5 days** | **10 days** |  | **2℃** | **5℃** | **10℃** |  | **0 days** | **90 days** | **150 days** |
| Northeast | 15 (-57, 207) | 39 (-50, 289) | 103 (18, 247) |  | 49 (32, 68) | 41 (16, 73) | 132 (52, 255) |  | -95 (-98, -90) | 134 (72, 218) | 126 (60, 219) |
| Inner Mongolia | 11 (-6, 32) | 11 (-26, 67) | -15 (-95, 1316) |  | 22 (8, 39) | 33 (13, 57) | 81 (4, 216) |  | -92 (-95, -86) | 63 (1, 161) | 150 (53, 309) |
| Northwest | 18 (-82, 673) | 11 (-78, 464) | 28 (-45, 197) |  | 36 (23, 50) | 39 (23, 58) | 69 (18, 142) |  | -91 (-94, -87) | 37 (-1, 90) | 26 (-3, 64) |
| North | 9 (-30, 71) | 2 (-38, 66) | 88 (34, 165) |  | 32 (22, 42) | 30 (17, 46) | 150 (54, 308) |  | -83 (-89, -75) | 151 (97, 220) | 70 (40, 106) |
| Huang-Huai-Yangtze | 1 (-9, 13) | 6 (-17, 36) | 49 (27, 76) |  | 29 (21, 38) | 32 (22, 43) | 223 (92, 444) |  | -87 (-91, -81) | 415 (314, 540) | 137 (99, 182) |
| Jiangnan | 3 (-6, 11) | 11 (-8, 35) | 29 (12, 48) |  | 32 (22, 43) | 32 (19, 47) | 239 (34, 757) |  | -88 (-91, -84) | 450 (338, 592) | 121 (82, 168) |
| South | -6 (-31, 30) | 1 (-31, 49) | 8 (-38, 86) |  | 19 (5, 34) | 12 (-6, 33) | 87 (-19, 329) |  | -52 (-66, -34) | 109 (77, 146) | 72 (34, 120) |
| Southwest | 30 (17, 44) | 25 (9, 43) | 11 (-30, 75) |  | 41 (30, 52) | 29 (17, 43) | 233 (-49, 2083) |  | -86 (-91, -80) | 163 (110, 230) | 165 (110, 233) |
| China mainland | 11 (7, 15) | 16 (11, 22) | 38 (27, 50) |  | 32 (27, 36) | 31 (26, 37) | 121 (85, 163) |  | -86 (-88, -83) | 188 (159, 220) | 98 (82, 117) |

**Table S4.** Spearman’s correlation coefficients between different sociodemographic factors.

| Sociodemographic  factors | Population  density | Population  size | GDP  per capita | Urbanization  rate | Population  growth rate | Hospital  beds | Collections | PM_2.5_ |
| --- | --- | --- | --- | --- | --- | --- | --- | --- |
| Population density | 1.00 |  |  |  |  |  |  |  |
| Population size | 0.57^a^ | 1.00 |  |  |  |  |  |  |
| GDP per capita | 0.40^a^ | 0.13 | 1.00 |  |  |  |  |  |
| Urbanization rate | 0.44^a^ | 0.12 | 0.83^a^ | 1.00 |  |  |  |  |
| Population Growth rate | 0.22^a^ | 0.15 | -0.07 | -0.03 | 1.00 |  |  |  |
| Hospital beds | -0.06 | -0.12 | 0.32^a^ | 0.32^a^ | -0.37^a^ | 1.00 |  |  |
| Collections | 0.16 | 0.00 | 0.74^a^ | 0.72^a^ | -0.08 | 0.28^a^ | 1.00 |  |
| PM_2.5_ | 0.38^a^ | 0.33^a^ | -0.04 | -0.02 | -0.08 | 0.04 | -0.25^a^ | 1.00 |

Note: ^a^*P*<.001; Population density (person/km^2^); Population size, Annual average population (persons); GDP per capita (Chinese yuan); Urbanization rate (%); Population growth rate, Natural population growth rate(‰); Hospital beds, Number of Beds of Hospitals (bed); Collections, Collections of Public Libraries per 100 Persons (copy); PM_2.5_, PM_2.5_ concentration (μg/m^3^).

**Fig. S5** The correlation between some sociodemographic factors and the relative risk of influenza caused by cold spells


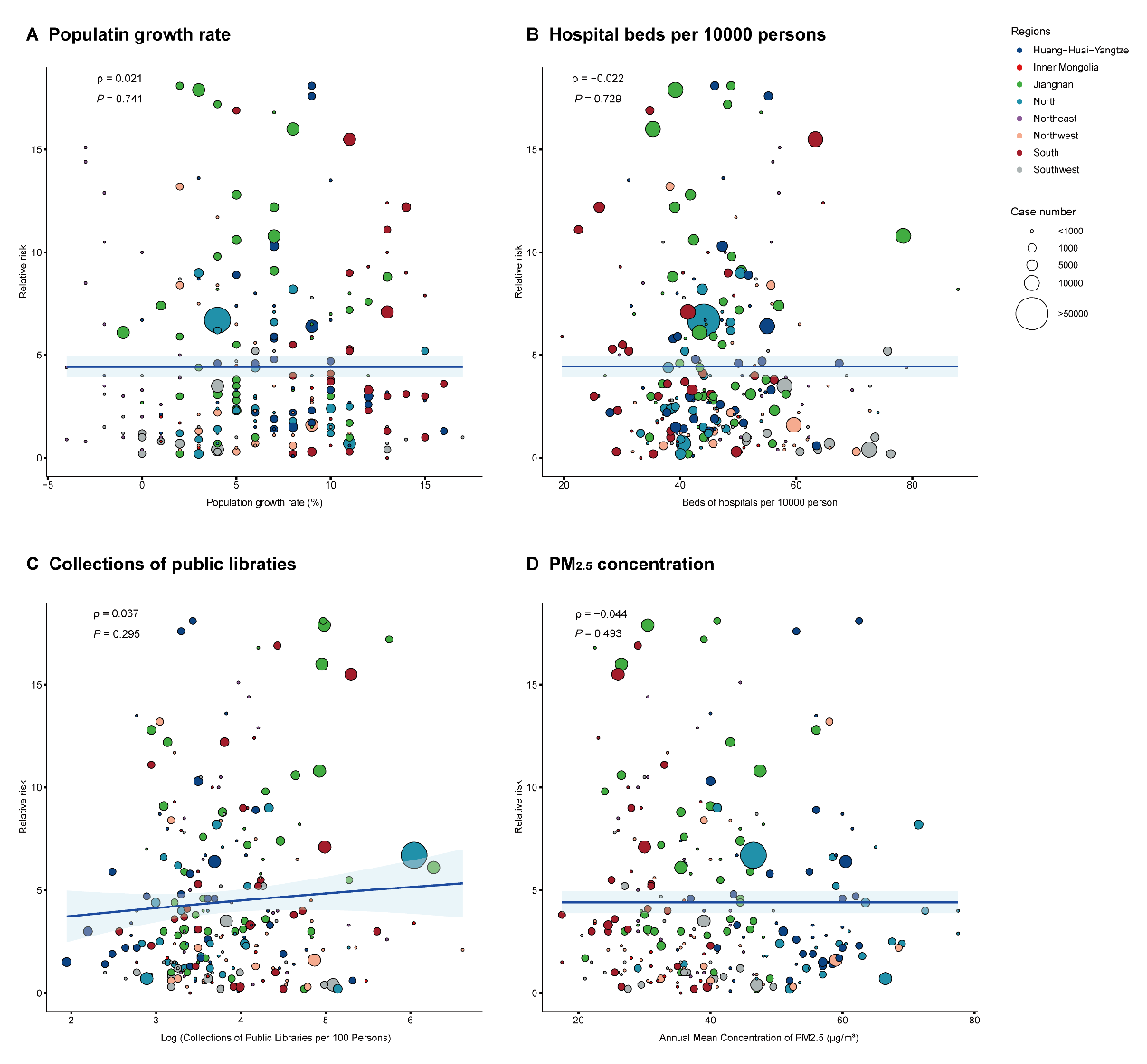


**Table S5.** Sensitivity analysis by changing the degrees of freedom (df) for temperature, maximum lag days, long-term trends and seasonality on the pooled overall and added effects under the optimum cold spell definition

|  | Df | | |  | RR (95% CI) | | |
| --- | --- | --- | --- | --- | --- | --- | --- |
|  | Temperature | maximum lag days | time |  | Overall effects |  | Added effects |
|  | 5 | 21 | 3 |  | 3.14 (2.71, 3.64) |  | 0.34 (0.21, 0.55) |
|  | 5 | 21 | 5 |  | 3.54 (3.06, 4.10) |  | 0.34 (0.21, 0.56) |
|  | 3 | 21 | 4 |  | 3.38 (2.93, 3.91) |  | 0.36 (0.23, 0.57) |
|  | 4 | 21 | 4 |  | 3.33 (2.88, 3.86) |  | 0.34 (0.22, 0.54) |
|  | 5 | 14 | 4 |  | 2.76 (2.49, 3.05) |  | 0.42 (0.29, 0.60) |
|  | 5 | 28 | 4 |  | 4.18 (3.43, 5.09) |  | 0.38 (0.21, 0.70) |
|  | 5 | 21 | 4 |  | 3.35 (2.89, 3.88) |  | 0.34 (0.21, 0.54) |

**Fig. S6** Sensitivity analysis by changing the df for temperature (A) and long-term trends and seasonality (B) on the percent changes in influenza incidence associated with the cold spells characteristics

**
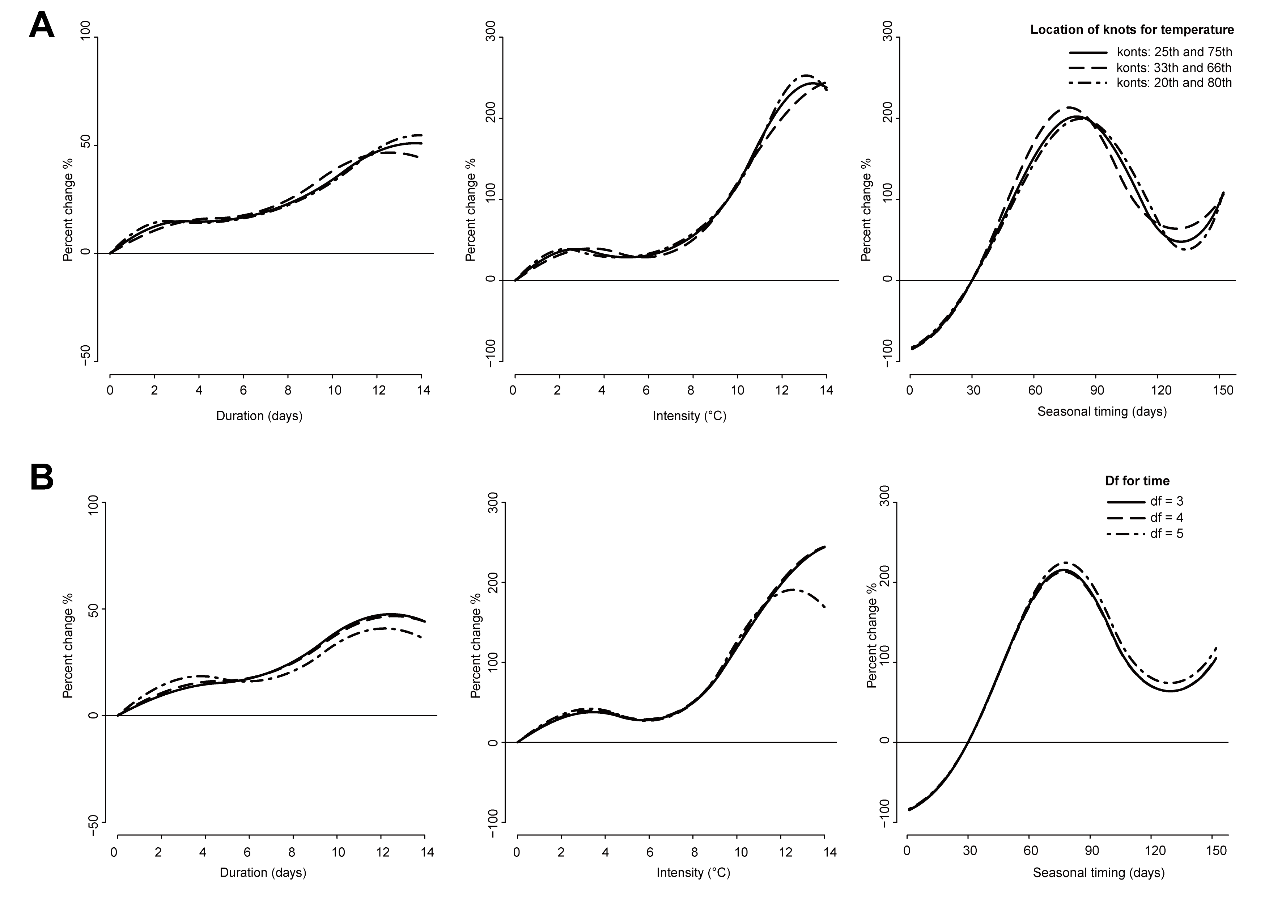
**

**Table S6.** Relationship between city-specific socioeconomic indicators and overall estimates of the association between cold spells and influenza incidence

| **Socioeconomic factors** | **Quartiles** |  | **P values for difference** | |
| --- | --- | --- | --- | --- |
| Population density | Q1 (Ref) |  | Unadjusted model | Adjusting population structure |
|  | Q2 |  | 0.387 | 0.459 |
|  | Q3 |  | 0.003 | 0.006 |
|  | Q4 |  | <0.001 | 0.001 |
| Population size | Q1 (Ref) |  |  |  |
|  | Q2 |  | 0.005 | 0.011 |
|  | Q3 |  | 0.021 | 0.028 |
|  | Q4 |  | <0.001 | 0.003 |
| GDP per capita | Q1 (Ref) |  |  |  |
|  | Q2 |  | 0.230 | 0.112 |
|  | Q3 |  | 0.629 | 0.456 |
|  | Q4 |  | 0.001 | 0.039 |
| Urbanization rate | Q1 (Ref) |  |  |  |
|  | Q2 |  | 0.157 | 0.064 |
|  | Q3 |  | 0.053 | 0.043 |
|  | Q4 |  | <0.001 | 0.008 |
